# Overexpression of an Orchid (*Dendrobium nobile*) *SOC1/TM3-Like* Ortholog, *DnAGL19*, in *Arabidopsis* Regulates *HOS1-FT* Expression

**DOI:** 10.3389/fpls.2016.00099

**Published:** 2016-02-09

**Authors:** Xiao-Ru Liu, Ting Pan, Wei-Qi Liang, Lan Gao, Xiao-Jing Wang, Hong-Qing Li, Shan Liang

**Affiliations:** Guangdong Provincial Key Laboratory of Biotechnology for Plant Development, School of Life Sciences, South China Normal UniversityGuangzhou, China

**Keywords:** *Dendrobium*, flowering, DnAGL19, vernalization, HOS1, FT, SOC1/TM3-like

## Abstract

Flowering in the appropriate season is critical for successful reproduction in angiosperms. The orchid species, *Dendrobium nobile*, requires vernalization to achieve flowering in the spring, but the underlying regulatory network has not been identified to date. The MADS-box transcription factor *DnAGL19* was previously identified in a study of low-temperature treated *D. nobile* buds and was suggested to regulate vernalization-induced flowering. In this study, phylogenetic analysis of DnAGL9 and the MADS-box containing proteins showed that DnAGL19 is phylogenetically closely related to the SOC1-like protein from orchid *Dendrobium* Chao Parya Smile, DOSOC1. The orchid clade closed to but is not included into the SOC1-1/TM3 clades associated with either eudicots or monocots, suggesting that DnAGL19 is an SOC1-1/TM3-like ortholog. *DnAGL19* was found to be highly expressed in pseudobulbs, leaves, roots, and axillary buds but rarely in flowers, and to be substantially upregulated in axillary buds by prolonged low-temperature treatments. Overexpression of *DnAGL19* in *Arabidopsis thaliana* resulted in a small but significantly reduced time to bolting, suggesting that flowering time was slightly accelerated under normal growth conditions. Consistent with this, the *A. thaliana APETELA1* (*AP1*) gene was expressed at an earlier stage in transgenic lines than in wild type plants, while the *FLOWERING LOCUS T* (*FT*) gene was suppressed, suggesting that altered regulations on these transcription factors caused the weak promotion of flowering. *HIGH EXPRESSION OF OSMOTICALLY RESPONSIVE GENE 1* (*HOS1*) was slightly activated under the same conditions, suggesting that the *HOS1-FT* module may be involved in the *DnAGL19*-related network. Under vernalization conditions, *FT* expression was significantly upregulated, whereas *HOS1* expression in the transgenic *A. thaliana* has a level similar to that in wild type. Taken together, these results suggest that *DnAGL19* controls the action of the *HOS1-FT* module depending on temperature cues, which could contribute to regulation of *D. nobile* flowering time. These data provide insights into how flowering is fine-tuned in *D. nobile* to acclimate the plant to seasonal changes in temperature.

## Introduction

Proteins containing the conserved MADS-box typically function as transcription factors and are present in a wide range of organisms, from fungi, slime molds, and metazoans to land plants. Putative DNA-binding MADS domains have also been found in bacteria (Mushegian and Koonin, 1996; Masiero et al., 2011; Smaczniak et al., 2012). In flowering plants, MADS-box proteins regulate diverse processes, including floral development, root growth, ovule and female gametophyte development, fruit ripening, and dehiscence (Zhang and Forde, 1998; Ng and Yanofsky, 2001; Giovannoni, 2004; Whipple et al., 2004; Colombo et al., 2008; Liu et al., 2009; Voogd et al., 2015). Two classes of MADS-domain proteins have been identified, based on the sequence of the MADS domain, and these have been named type I (SRF-like) and type II (MEF2-like; Masiero et al., 2011; Smaczniak et al., 2012).

SUPPRESSOR OF OVEREXPRESSION OF CO 1 (SOC1) and SOC1/TM3-like proteins are type II MADS-box proteins. They contain the highly conserved MADS-box, K domain, and a SOC1-motif at the C-terminus (Vandenbussche et al., 2003). In *Arabidopsis thaliana*, these proteins comprise a small subfamily that includes AGL20 (SOC1), AGL19, AGL14, AGL42, AGL71, and AGL72. *A. thaliana* SOC1 integrates the signals from photoperiodism, prolonged low-temperatures (vernalization), and the gibberellin and autonomous pathways, and controls the expression of the *LEAFY* (*LFY*) gene to promote floral initiation (Liu et al., 2008; Lee and Lee, 2010). It is also involved in prevention of a perennial-type lifestyle (Melzer et al., 2008), and plays a role in crosstalk between cold sensing and flowering (Seo et al., 2009). AGL19 also acts as a flowering activator, functioning in vernalization-related floral transition (Schönrock et al., 2006), while AGL42, AGL71, and AGL72 promote floral transition in axillary meristems (Dorca-Fornell et al., 2011). AGL14 was recently demonstrated to be involved in both shoot apical meristem transition and in the regulation of the *AP1* and *TERMINAL FLOWER 1 (TFL1)* genes in *A. thaliana* (Perez-Ruiz et al., 2015). *SOC1/TM3-like* genes have been identified in a wide range of plant species, such as the *TrcMADS1* gene from *Trillium camtschatcense* (*Trilliaceae*) (Nakamura et al., 2005), the *GhSOC1* and the *GhSOC2* gene from *Gerbera hybrida* (Ruokolainen, 2011), the *PsSOC1* gene from *Paeonia suffruticosa* (tree peony) (Zhang et al., 2014) and the *DOSOC1* gene from the orchid *Dendrobium* Chao Parya Smile (Ding et al., 2013). Most SOC1/TM3-like proteins play roles during the phase change from vegetative to reproductive development. For example, the rice SOC1 ortholog, OsMADS50, promotes flowering when overexpressed in either *A. thaliana* or rice, while loss of function of *OsMADS50* in rice causes delayed flowering under long-day photoperiod (LD) conditions (Tadege et al., 2003; Lee et al., 2004; Ryu et al., 2009). In addition to flowering regulation, SOC1/TM3-like orthologs can play roles in other biological processes. For example, combined mutations at *SOC1* and *FUL* loci in *A. thaliana* were reported to result in renewed growth at sites with dead cauline leaves, suggesting that SOC1 promotes the maintenance of an annual life-style (Melzer et al., 2008). In another study, constitutive expression in *Gerbera* of the *A. thaliana AGL71* and *AGL72* ortholog, *GhSOC1*, did not affect flowering time but led to partial loss of floral organ identity (Ruokolainen, 2011). In addition, *FvSOC1* from the perennial short-day plant, woodland strawberry (*Fragaria vesca*) was shown to promote vegetative growth but not flowering (Mouhu et al., 2013).

The *SOC1* gene regulatory network (GRN) has been extensively studied in *A. thaliana*, where *SOC1* is regulated by prolonged low-temperature (vernalization) through the *FLOWERING LOCUS C* (*FLC*)-dependent pathway and transcription of *SOC1* is enhanced due to the suppression of *FLC* gene (Lee and Lee, 2010). *SOC1* expression is also up-regulated by the zinc finger transcription factor CONSTANS (CO; Lee and Lee, 2010). *LFY* is a downstream target of SOC1 (Liu et al., 2008; Lee and Lee, 2010), and is activated by the SOC1-AGL24 complex to up-regulate the expression of the *AP1* gene. The SOC1 protein not only directly targets to its own gene, but also to those of other flowering time regulators, some of which act upstream of SOC1 (Immink et al., 2012). For example, SOC1 physically interacts with AP1 to form a higher order complex and suppress its own transcription (Immink et al., 2012).

In addition to controlling flowering, SOC1 also regulates other processes, such as the activation *CBF* or *COR* genes upon cold stress, suggesting that SOC1 serves as a hub for both cold-induced responses and floral development (Seo et al., 2009). It is known that cold affects flowering and that vernalization accelerates the flowering of *A. thaliana* and other temperate species (Sung and Amasino, 2005; Kim et al., 2009). In contrast, low ambient temperature (e.g., 16°C) or intermittent cold exposure (e.g., 4°C for 6 h/day) delays flowering (Kim et al., 2004; Seo et al., 2009). This has been associated with a ubiquitin E3 ligase protein, encoded by *HIGH EXPRESSION OF OSMOTICALLY RESPONSIVE GENE 1* (*HOS1*), which mediates the degradation of CO during cold conditions, resulting in transcriptional suppression of *FT* (Jung et al., 2012; Lazaro et al., 2012). HOS1 can also act as a competitor to dissociate the histone deacetylase HDA6 from MULTICOPY SUPPRESSOR OF IRA1 4 protein (MSI4, also known as FVE), thereby releasing *FLC* from transcriptional inhibition (Jung et al., 2013). The loss-of-function *HOS1* mutant, *hos1-1*, flowers early even under normal conditions, and some floral regulators, such as *FT*, are expressed at higher levels in this mutant, demonstrating that the HOS1-FT module contributes to flowering regulation (Ishitani et al., 1998; Jung et al., 2012; Lazaro et al., 2012).

*Dendrobium* is a genus in the *Orchidaceae* family with more than 1,200 species (Adam, 2011), many of which are valued for their use in herbal medicine and ornamental gardening. Cold temperatures have different effects on flowering in different *Dendrobium* orchids. Generally, the Nobile-type *Dendrobium* can adapt to long-term low-temperatures in winter and bloom along the cane at each node in spring (Siam Orchid Culture Co., Ltd.^1^), while the Cane-type *Dendrobium* species bloom in autumn before the weather turns cold. *SOC1/TM3-like* genes were identified from *Dendrobium* orchids previously. *DOSOC1* is a *SOC1* ortholog from *Dendrobium* Chao Parya Smile. It is predominantly expressed in reproductive organs and promotes flowering when overexpressed in either *A. thaliana* or *Dendrobium* Chao Parya Smile. Additionally, *DOSOC1* affects flower morphogenesis by abolishing the development of flower buds in transgenic *Dendrobium* Chao Parya Smile plants (Ding et al., 2013). DnAGL19 was identified from the Nobile-type orchid *Dendrobium nobile* Lindl. It is closely related to members of *A. thaliana* SOC1/TM3-like subfamily and was proposed to function in flowering regulation network by acting upstream of the *D. nobile AP1* ortholog(s) under prolonged low temperature (Liang et al., 2012). It is not clear by now whether and how these orchid SOC1/TM3-like proteins play roles in cold acclimation and the cold-associated flowering control. In this current study, we investigated the roles of DnAGL19 in flowering regulation under normal growth condition and in response to vernalization, aiming at exploring the genetic link between DnAGL19 and other components of the regulatory network and the mechanism by which DnAGL19 controls flowering.

## Materials and Methods

### Plant Material and Growth Conditions

*Dendrobium nobile* plants were grown in a greenhouse under natural conditions without regulation of photoperiod at the Orchid Research Center at South China Normal University. For vernalization treatments, adult plants were exposed to low temperatures for 0, 5, 10, 20, or 30 days at 16°C/10°C (day/night). Axillary buds were collected from each node of the pseudobulbs and pooled. Each independent biological sample was comprised of axillary buds from four to five plants and at least three biological replicates for each treatment were analyzed. Untreated control plant material was collected in parallel. Samples for detection of organ specific expression were collected from juvenile (young) and adult (old) plants that had not undergone vernalization treatment.

*Arabidopsis thaliana* (Col ecotype) was used to generate transgenic plants. Seeds were sterilized and sowed on MS (Murashige and Skoog) medium supplemented with 3% (w/v) sucrose and 0.8% (w/v) agar. Controlled environmental conditions were provided in growth chambers at 22 ± 1°C and 50–70% relative humidity. Plants were illuminated with cool-white fluorescent lights. LD conditions consisted of 16 h of light followed by 8 h of darkness. For vernalization treatments, seedlings were transferred to darkness and held at 4°C for 4 weeks after growing under normal LD conditions for 1 day. Seedlings were cultured under normal LD conditions after vernalization and sampled at the indicated time points.

### Generation of Transgenic *A. thaliana*

The complete *DnAGL19* open reading frame (KU373056) (Liang et al., 2012) was amplified from *D. nobile* cDNA that is reversely transcribed from total RNA extracted from axillary buds, followed by cloning into the pMD20-T vector (TaKaRa, Dalian, China) to sequence (Invitrogen, Shanghai, China). Primers for the full-length ORF amplification are listed in Supplementary Table S1. The *DnAGL19* coding sequence (636-bp, with the stop codon removed) was inserted into the pCanG-Myc vector to generate the *CaMV 35S::DnAGL19-6Myc* construct, in which a sequence encoding six tandem repeats of a Myc-tag was fused to the 3′-end of the *DnAGL19* ORF. The *35S::DnAGL19-6Myc* construct was transformed into *A. thaliana* seedlings using the floral-dip method (Clough and Bent, 1998), and homozygote lines were selected for further analysis.

### Measurement of Flowering Time

Flowering time was recorded using two parameters: the number of rosette leaves at flowering (RL), and days from sowing to bolting (DTB). For the number of RL, only those juvenile rosette leaves with trichomes on the adaxial surface were counted at the moment of the first flower opening. Adult rosette leaves with both adaxial and abaxial trichomes were not recorded. For the DTB, the days needed from sowing to the first flower bud being visible were recorded. At least 12 plants were measured for each condition and the data are shown as mean ± SD. Statistical significance was determined using the Student’s *t*-test.

### RT-PCR and Real-Time Quantitative PCR

Total RNA was extracted from *D. nobile* and *A. thaliana* seedlings using the E.A.N.A. Plant RNA Kit (OMEGA BIO-TEK, USA) and reversely transcribed to generate cDNA using the PrimeScript^TM^ Reverse Transcriptase (TaKaRa, Dalian, China) according to the manufacturer’s instructions. RT-PCR was used to detect organ specific expression patterns and real-time quantitative PCR was used for a time-course analysis of expression following vernalization in *D. nobile*. *D. nobile 18S rRNA* was used as a reference control. Gene-specific primer pairs are shown in Supplementary Table S1. The qPCR reactions and thermal program are as described below.

Real-time qPCR was used to quantify the expression levels of flowering associated genes in transgenic *A. thaliana*. A 25 μl qPCR reaction contains cDNA that reversely transcribed from 25 ng total RNA, specific primers with final concentration of 200 nM for each and 12.5 μl THUNDERBIRD SYBR qPCR Mix (TOYOBO, Japan) that contains the Taq DNA polymerase and the florescent dye SYBR Green. Real-time qPCR was run in an ABI PRISM1 7500 system (Applied Biosystems, USA) following the program of a pre-denaturation for 1 min at 95°C and 40 cycles of 15 s at 95°C and 45 s at 60°C. Specific primers are listed in Supplementary Table S1. Relative expression levels for a given gene under the indicated condition were calculated using the 2^-ΔΔCt^ method by normalizing to the *A. thaliana ACTIN2/7* gene and calibrating to the wild type sample. Three biological samples and triplicate qPCR reactions for each combination of primers and sample were analyzed.

### Sequence Analysis

Sequences used in the alignment and phylogenetic analysis were retrieved from the databases at Phytozome v10.2^2^ or NCBI^3^, based on BlastP searches using the deduced peptide sequence of *DnAGL19* as a query. Databases incorporating a total of 22 species were searched at Phytozome v10.2, including: (1) the dicotyledons *A. lyrata* (Aly), *A. thaliana* (At), *Aquilegia coerulea* Goldsmith (Aquca), *Boechera stricta* (Bostr), *Brassica rapa* FPsc (Brara), *Capsella grandiflora* (Cagra), *Capsella rubella* (Carubv), *Cucumis sativus* (Cucsa), *Medicago truncatula* (Medtr), *Citrus sinensis* (Orange), *Solanum tuberosum* (PGSC), *Populus trichocarpa* (Potri), *Theobroma cacao* (Thecc) and *Eutrema salsugineum* (Thhalv); (2) the monocotyledonous grasses *Oryza sativa* (Os), *Brachypodium distachyon* (Bradi), *Zea mays* (GRMZM), *Panicum virgatum* (Pavir) and *Sorghum bicolor* (Sobic); (3) the Pteridophyta species *Selagenella moellendorffii;* (4) the moss *Physcomitrella patens;* and (5) the Chlorophyte *Chlamydomonas reinhardtii*. The phylogenetic relationships of all mentioned species are shown in Supplementary Figure S1 (adopted from Phytozome v10.2). The DOSOC1 (AGK07583) sequence was retrieved from NCBI. Alignment and phylogenetic analysis, as well as tree editing, was carried out using MEGA6 (Tamura et al., 2013) or ClustalX2 (Larkin et al., 2007). Conserved sites in the aligned sequences, defined as those with more than 70% similarity, were highlighted using BOXSHADER 3.21^4^ and WebLoGo^5^ was used to generate the consensus sequences of the MADS-box, K-domain, and the SOC1-motif.

## Results

### *DnAGL19* Encodes a SOC1/TM3-Like Ortholog

We previously proposed that *DnAGL19* encodes a protein closely related to the *A. thaliana* AGL19 (Liang et al., 2012). To further elucidate the evolutionary relationship between DnAGL19 and its homologs, protein sequences were retrieved from a wide range of species by BlastP searches using the deduced amino acid sequence of *DnAGL19* as a query and an *E*-value cut-off of <10^-45^. This yielded a total of 151 sequences from nineteen representative angiosperm species. Initially, no homologs were found for the Pteridophyte species *S. moellendorffii*, the moss *P. patens* and the Chlorophyte *C. reinhardtii*; however, when less stringent criteria were used to re-search the databases (only the amino acids 1–70 of the MADS domain of DnAGL19 and an *E*-value of <10^-10^) we identified one protein sequence from *C. reinhardtii* containing a unique MADS domain at the N-terminus, nineteen protein sequences from *P. patens* and ten from *S. moellendorffii*. The top matched entries from these three species were used for further analysis.

The complete sequences of DnAGL19, DOSOC1, and all 154 homologs were aligned and subjected to a phylogenetic analysis. Using the MADS-containing protein sequence from *C. reinhardtii* as the out-group, a neighbor joining (NJ) tree was constructed using 1,000 bootstrap replications (Supplementary Figure S2). This indicated that the *P. patens* and *S. moellendorffii* sequences, as well as the angiosperm homologs of OsMADS6, a protein similar to *A. thaliana* AGL6, clustered in a clade that was independent of the SOC1/TM3-like clade (Supplementary Figure S2), suggesting that the SOC1/TM3-like proteins from all the tested angiosperm species originated from a common ancestor and are closely related to the AGL6-like proteins.

**FIGURE 1:**
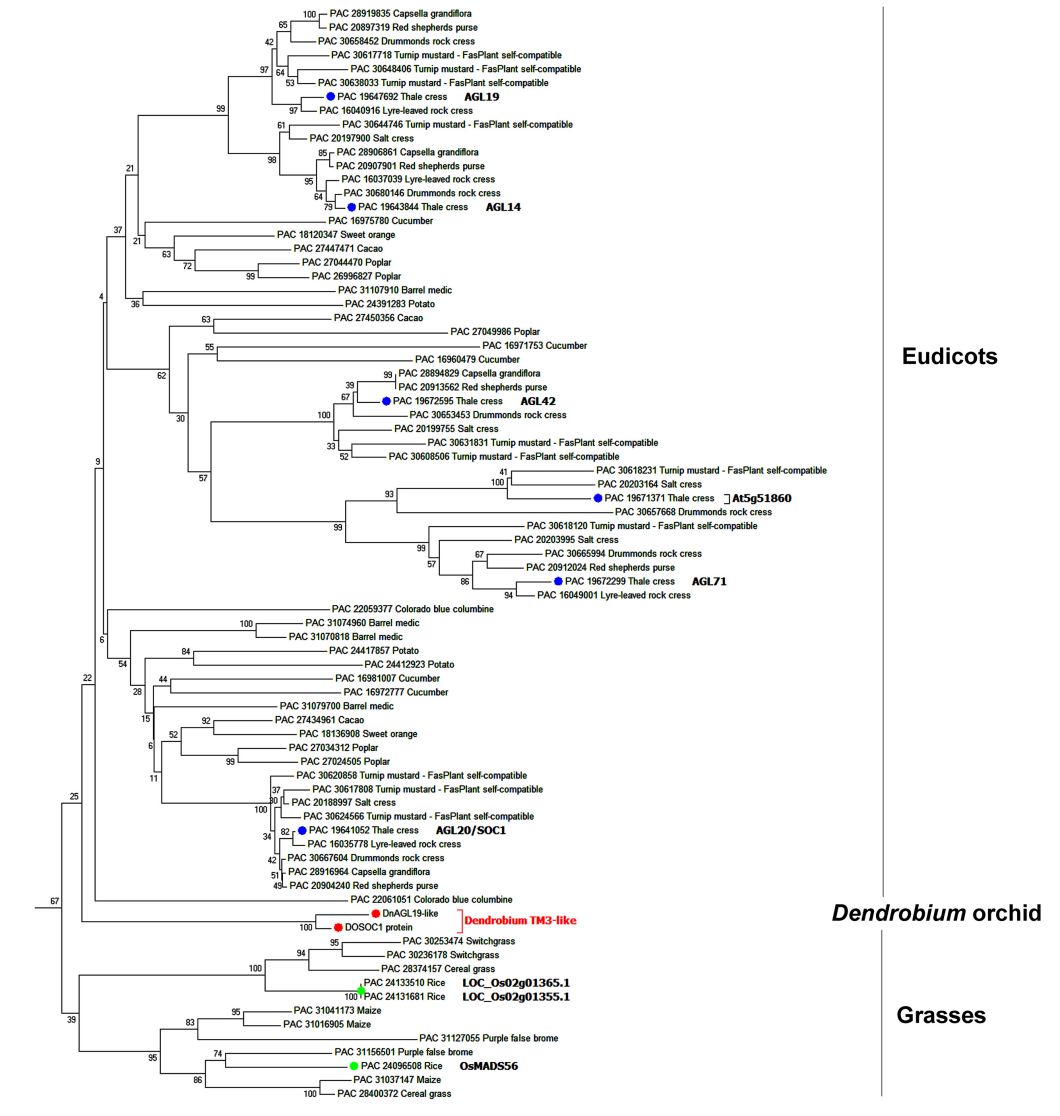
**Phylogenetic analysis of DnAGL19 and its homologs**. Phylogenetic analysis of DnAGL19, DOSOC1, and homologous proteins was performed using MEGA6. A preliminary neighbor joining (NJ) tree was constructed using the *Chlamydomonas reinhardtii* MADS-box protein, PAC 30775785 (accession No. at Phytozome 10.2), as the out-group. The subtree containing the SOC1/TM3-like proteins is shown here. Orthologs from eudicots and monocot grasses are grouped independently, and both groups are independent from the *Dendrobium* SOC1/TM3-like branch (red). SOC1/TM3-like proteins from *A. thaliana* and rice (*Oryza sativa*) are shown in green and blue, respectively. Proteins are indicated by “Accession No. at Phytozome 10.2 + species common name.” DnAGL19 and DOSOC1 are orchid SOC1/TM3-like proteins from *Dendrobium nobile* and *Dendrobium* Chao Parya Smile, respectively.

**FIGURE 2:**
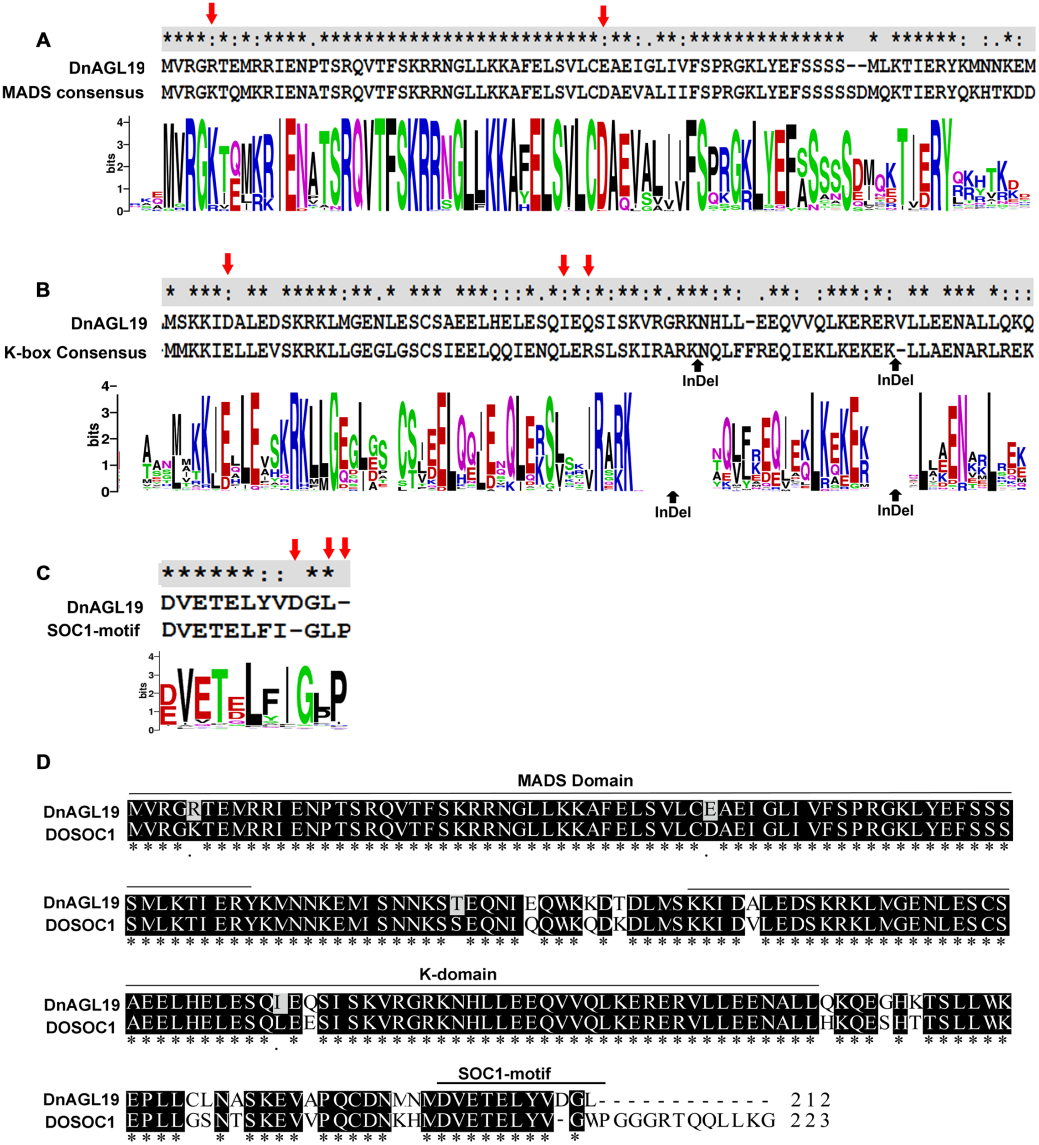
**Alignment of DnAGL19 to DOSOC1 and consensus sequences of the conserved domains**. Consensus sequences of the MADS-box **(A)**, K-domain **(B)** and SOC1-motif **(C)** of SOC1/TM3-like proteins were generated by WebLoGo based on an alignment of DnAGL19, DOSOC1 and 151 SOC1-motif-containing proteins. The consensus sequences are shown above the graphs to align to corresponding domains on DnAGL19. “^∗^” indicates the conserved sites. In panel **(B)**, two indel sites are indicated by upward pointing arrows. Panel **(D)** shows the alignment of DnAGL19 to DOSOC1. Identical sites are shaded. The variations within the MADS Box, the K domain and the SOC1-motif are indicated by red downward pointing arrows in panel **(A–C)**.

Another NJ tree was generated with the sequences from the same species removed if they grouped together with bootstrap value of >99% in the preliminary tree. This simplified tree had a similar topological structure to the preliminary tree, and we then focused on the subtree containing the SOC1/TM3-like subfamily (**Figure 1**). Three angiosperm SOC1/TM3-like protein branches were clearly recognized, with the orchid SOC1/TM3-like proteins, DnAGL19 and DOSOC1, clustered together into a branch that was independent from both the eudicot and the monocot branches (**Figure 1**). An alignment of DnAGL19 and DOSOC1 with orthologous angiosperm proteins showed that DnAGL19 contains the conserved MADS-box, K-domain, and SOC1-1 motif, with overall identities of 78, 59, and 73% to the consensus sequences, respectively (Supplementary Figure S3). In addition, amino acid substitutions were observed between DnAGL19 and DOSOC1 at two positions within the MADS-box (R5K, E40D), three within the K-domain (A103V, I131L, Q133E) and thirteen scattered in the intervals between the conserved domains (**Figure 2**, Supplementary Table S3). All substitutions within the MADS and K domains of DnAGL19 were rarely presented on the sequences of the tested SOC1/TM3-like proteins (**Figure 2**, Supplementary Table S3). For example, the Arg at the fifth position from the N-end of MADS-box in DnAGL19 was only shared with ∼9% of the tested SOC1/TM3-like proteins. Variations were also found at the final three positions of the SOC-motif. In comparing with the consensus sequence, the DnAGL19 had an insertion of “Asp (D)” before the highly conserved “Gly (G)” at the antepenult position and a deletion of the conserved “Pro (P)” at the final position in the SOC1-motif, while DOSOC1 showed the “Trp (W)” substitute for the “Leu (L)” at the penultimate position (**Figure 2**). As a result, “DGL” was presented at the final three positions of this motif on DnAGL19 instead of “GWP” on DOSOC1 (**Figure 2D**).

### *DnAGL19* Expression *In Planta*

**FIGURE 3:**
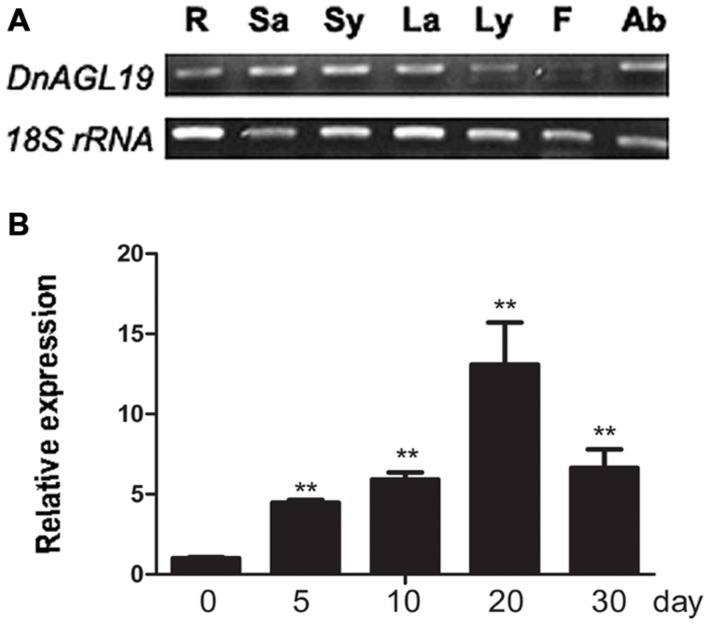
***In planta* expression of *DnAGL19*. (A)** Organ-specific expression of *DnAGL19* is shown. R, root; Sa: adult pseudobulbs; Sy: juvenile pseudobulbs; La: adult leaves; Ly: juvenile leaves; F: flowers; Ab: axillary buds. Total RNA was extracted from the indicated organs and RT-PCR was performed. *18s rRNA* was used as the endogenous control. **(B)** Time-course dynamics of the expression of *DnAGL19* during vernalization. Total RNA was extracted from axillary buds of vernalized *D. nobile*. The treating time duration is shown as days post start of vernalization. Three technical and biological replicates were analyzed by qPCR. Values are shown as mean ± SD. *18s rRNA* was used as the endogenous control and the “0”-day sample was used to calibrate the other samples. “^∗∗^” indicates *t*-test *p*-value < 0.01.

To determine the *DnAGL19* expression pattern in *D. nobile*, total RNA was extracted from various organs, including roots, pseudobulbs, leaves, flowers, and axillary buds. RT-PCR was performed using *18S rRNA* as the endogenous control, and *DnAGL19* expressed at higher levels in roots, pseudobulbs, leaves and axillary buds than in flowers (**Figure 3A**). *DnAGL19* transcript levels were also evaluated in vernalized *D. nobile* axillary buds that had been exposed to low temperatures. The cold treatment was performed in three consecutive years, and samples were harvested after 5, 10, 20, and 30 days of treatment. The qPCR analysis indicated that *DnAGL19* expression was induced by 5 days treatment and peaked after 10 or 20 days. Although slight differences were observed between biological replicate samples from different years, the trends over the time course were similar (**Figure 3B**). These results are consistent with a previous report (Liang et al., 2012), confirming that the *DnAGL19* gene expression is induced in axillary buds by prolonged low temperature treatments.

### Phenotypes of *DnAGL19* Overexpressing *A. thaliana*

**FIGURE 4:**
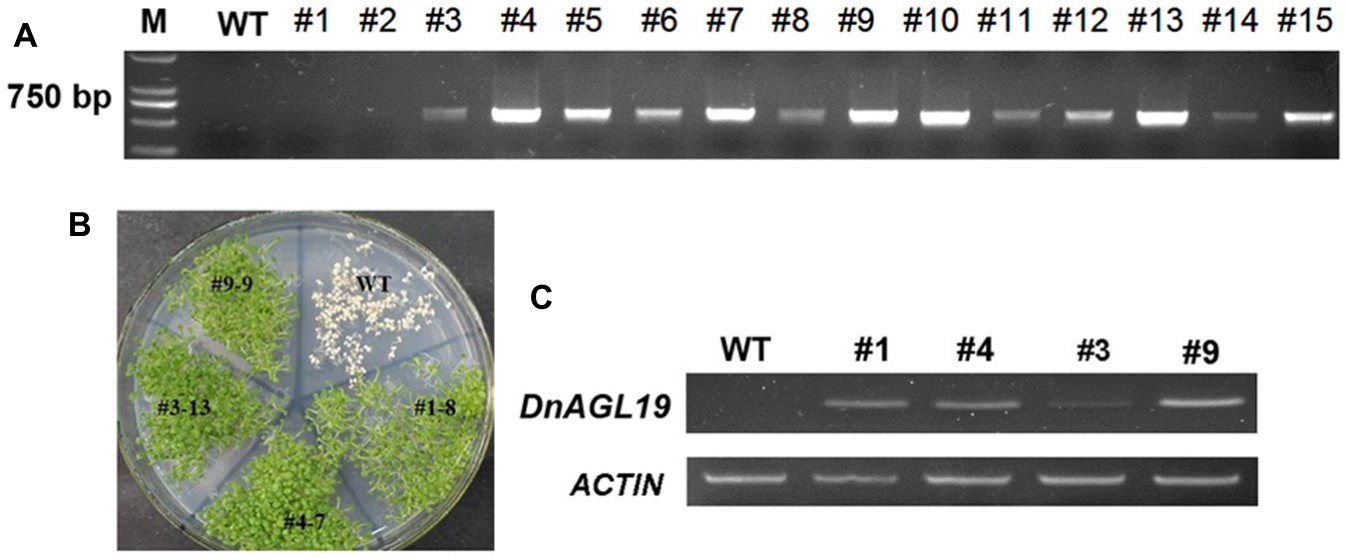
**Screening for homozygous *DnAGL19* overexpressing *Arabidopsis thaliana* lines. (A)** Detection of the *DnAGL19* insertion in putative transgenic lines. Genomic DNA was extracted from wild type *A. thaliana* and putative transgenic lines marked #1 to #15. The *DnAGL19* sequence (639 bp) was amplified using genomic DNA as a template. **(B)** Growth of identified homozygous plants on a MS plate containing 50 mg/L kanamycin. Lines # 1, # 3, # 4, # 9 and wild type are shown. **(C)** Expression of *DnAGL19* in transgenic lines. Total RNA was extracted from lines # 1, # 3, # 4, # 9 and wild type seedlings and reversed transcribed. RT-PCR was performed to detect the expression level of *DnAGL19*.

Phylogenetic analysis indicated that DnAGL19 is the ortholog of DOSOC1 and *A. thaliana* SOC1/TM3-like proteins (**Figure 1**) and so we next investigated whether and how DnAGL19 is involved in the regulation of flowering time. Transgenic *A. thaliana* plants harboring a 35S::*DnAGL19*-6*Myc* fusion construct were generated and the *DnAGL19* insertions were verified for 13 selected T_1_ plants by PCR (**Figure 4**). Of these, six lines were identified as harboring single-copy insertions (Supplementary Table S2), and four homozygous lines were finally obtained. Expression of *DnAGL19* in these homozygotes was detected using RT-PCR and we observed that lines OX-6myc # 1, 4, and 9 accumulated high levels of *DnAGL19* transcripts (**Figure 4**). Lines #4 and #9 were used for subsequent phenotypic analysis.

**FIGURE 5:**
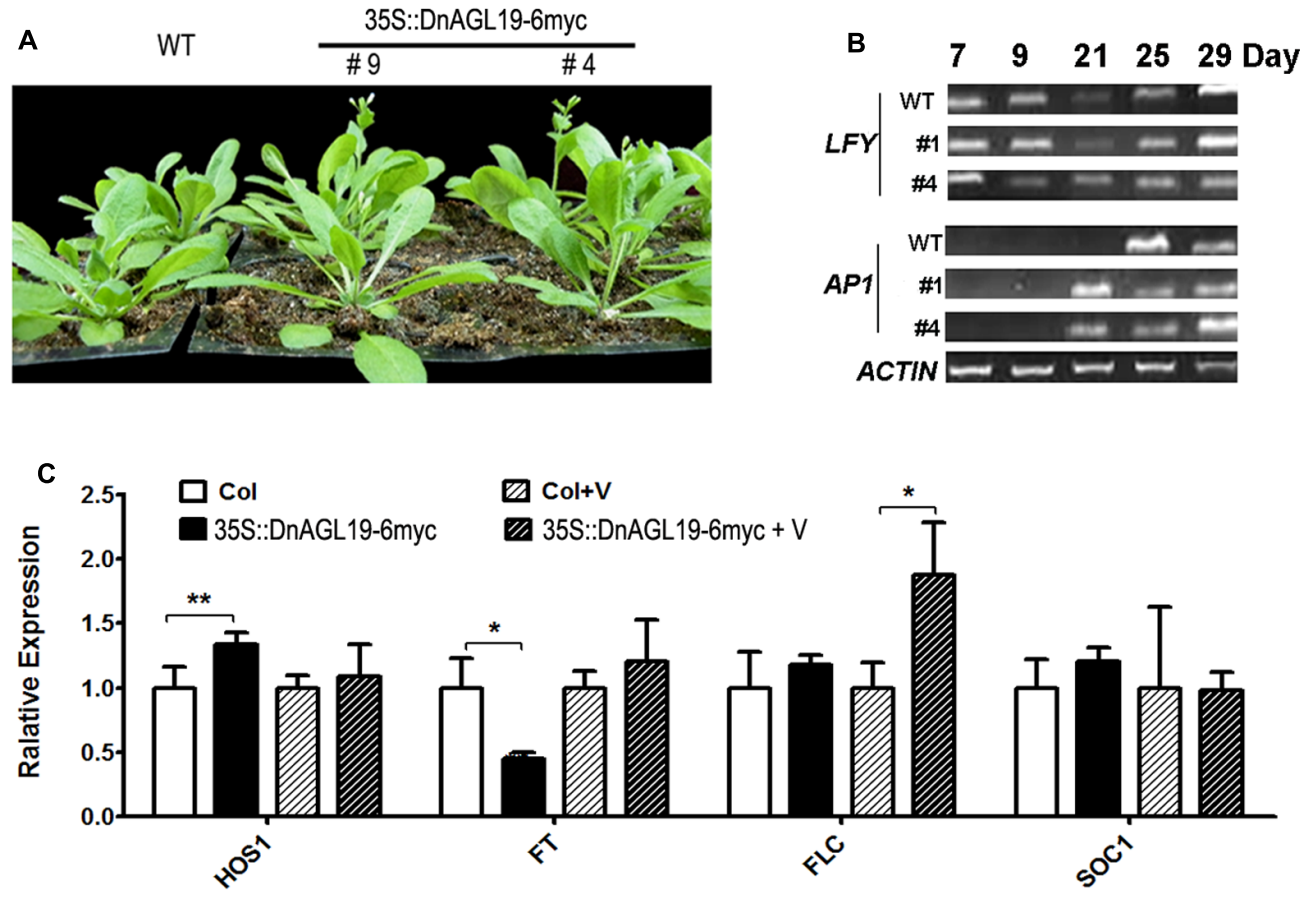
**Phenotypes of *Arabidopsis thaliana* lines overexpressing *DnAGL19*. (A)** Growth of 25-day old wild type (WT) and *DnAGL19* overexpressing lines (35S::DnAGL19-6myc #9 and #4) under normal LD condition. **(B)** Expression of *LFY* and *AP1* at different developmental stages in wild type and *DnAGL19* overexpressing lines (#1 and #4). Total RNA was extracted from seedlings of wild type and the overexpressing lines growing under LD conditions. RT-PCR was carried out. The developmental stages are shown as days after sowing. **(C)** Expression of flowering time associated genes (*HOS1, FT, FLC*, and *SOC1*) in wild type and *DnAGL19* overexpressing seedlings before and after vernalization. “N” indicates the non-vernalized plants and “V” indicates the vernalized ones. Total RNA was extracted from 10-day old seedlings. Real-time qPCR was carried out in triplicates for each sample/gene combination, and data are shown as mean ± SD, with ^∗^*p* < 0.05 and ^∗∗^*p* < 0.01.

Flowering phenotypes were recorded for the *DnAGL19* overexpressing lines grown under normal LD conditions or following vernalization (4°C for 4 weeks), with wild type *A. thaliana* serving as the control. As indicated in **Table 1**, the number of rosette leaves (RL) in the transgenic lines # 4 and # 9 was not statistically different from the wild type under normal conditions. However, the number of days from sowing to bolting (DTB), was significantly reduced by 2.0 and 1.4 days for line #4 and #9, respectively (**Table 1**, **Figure 5**), indicating a small acceleration of flowering in the *DnAGL19* overexpressing lines. We observed that the vernalization (V) counteracted this promotion to some extent, since the transgenic lines required a longer time to bolt, resulting in no statistical differences in DTB between transgenic lines and wild type plants (**Table 1**). These observations indicate that *DnAGL19* expression suppressed floral development as a consequence of vernalization in the transgenic lines. Flowering time was also evaluated at T2 generation for a population of *DnAGL19* overexpressing plants and the observations were similar to those for the homozygous lines #4 and #9 (Supplementary Figure S4).

**Table 1:** Flowering phenotypes of *DnAGL19* overexpressing *Arabidopsis thaliana.*

Genotype	Long-day (LD)		Vernalization (V, 4°C, 4 weeks)		
	DTB	RL	DTB	Diff.	RL
Wild type	31.5 ± 1.3	8.9 ± 1.1	45.5 ± 1.7	14.0	7.3 ± 1.3
OX-6myc #4	29.5 ± 0.8^∗∗^	9.3 ± 0.7	45.7 ± 1.1	16.2	7.8 ± 1.1
OX-6myc #9	30.1 ± 0.6^∗∗^	9.5 ± 0.5	46.4 ± 1.4	16.3	8.0 ± 1.0

*DTB, days from sowing to bolting; the 4-weeks of vernalization are included in DTB; RL, number of rosette leaves; Diff. = (DTB after vernalization) – (DTB under LD condition). Differences between overexpression lines and wild type were evaluated using *t*-tests, with ^∗∗^ indicating *p* < 0.01*.

### Expression of Flowering-Associated Genes in *DnAGL19* Overexpressing Lines Under LD Conditions

To determine the cause of the phenotypic differences between the transgenic lines and the wild type plants, the transcript levels of *LFY, AP1, SOC1, FT*, and *FLC* were evaluated in line #4. We observed that the *AP1* transcript abundance increased substantially in 21-day-old transgenic lines, which was earlier than in the wild type (**Figure 5B**). Real-time qPCR analysis demonstrated that this activation of *AP1* expression occurred even earlier, in 7-day-old plants (Supplementary Figure S6). However, the expression of *LFY* and *FT*, regulatory genes that act upstream of *AP1* (Wigge et al., 2005), did not correlate with the transcriptional activation of *AP1*. Transcription of *LFY* was not altered by overexpression of *DnAGL19* (**Figure 5B**, Supplementary Figure S6), indicating that *AP1* activation could not be attributed to *LFY* transcription. Furthermore, *FT* was down-regulated by 2.3-fold, on average, which was significantly different from the wild type (**Figure 5**), indicating that *FT* did not contribute to the activation of *AP1*. We therefore concluded that *DnAGL19* operates in an alternative pathway to activate *AP1* and promote flowering in *A. thaliana*. In addition, the expression levels of *SOC1* and *FLC* showed no dramatic differences between line #4 and wild type under LD conditions.

### Expression of Flowering Associated Genes in *DnAGL19*-Overexpression Lines in Response to Vernalization

Although the RL numbers in the *DnAGL19* overexpressing plants did not differ from those of the wild type, the significant difference of DTB under normal LD conditions disappeared after vernalization treatment (**Table 1**). In addition, the suppression of *FT* by overexpression of *DnAGL19* under non-vernalization conditions was not apparent following vernalization. The abundance of *FT* transcript returned to a level similar to that in wild type plants (**Figure 5**, Supplementary Figure S7); however, *FLC* expression was also 1.88-fold higher, significantly different from that in wild type plants. Together, these results suggested that the combined effects of vernalization and *DnAGL19* expression modulated the expression of both floral activator (e.g., *FT*) and inhibitor (e.g., *FLC*) genes.

### Expression of *HOS1* in *DnAGL19* Overexpressing *A. thaliana*

Our results indicated that overexpression of *DnAGL19* suppressed the expression of *FT* under normal growth condition. We therefore investigated the expression of the known upstream that targets the CO-FT module. As mentioned above, *HOS1* encodes an E3 ligase that is involved in cold-associated flowering regulation via the action of the CO-FT module (Ishitani et al., 1998; Jung et al., 2012; Lazaro et al., 2012). Interestingly, we observed that even under normal LD conditions the transcription of *HOS1* was substantially elevated in 9-day-old *DnAGL19* overexpressing plants compared to wild type, suggesting that a high level of DnAGL19 promotes the expression of *HOS1* (**Figure 5**, Supplementary Figure S7). After vernalization, the accumulation of the *HOS1* transcript in *DnAGL19* overexpressing lines returned to a level similar to that in wild type plants (**Figure 5**, Supplementary Figure S7).

## Discussion

SOC1/TM3-like homologs have previously been identified from diverse species, and as mentioned in the Introduction section, these proteins are functionally divergent among paralogs and across species. To date there have been few reports of SOC1/TM3-like proteins in orchid species. Ding et al. (2013) identified a SOC1 ortholog, named DOSOC1, from *Dendrobium* Chao Parya Smile and demonstrated that it functioned as a flowering activator and also played a role in floral organ development. We previously observed that *DnAGL19*, encoding a putative *D. nobile SOC1/TM3-like* protein, was activated under prolonged low-temperature treatments (Liang et al., 2012), and in this current study, we extended the investigation to the biological functions of *DnAGL19* to shed light on flowering regulation in *D. nobile*.

### DnAGL19 may have Functionally Diverged from DOSOC1 and Other Orthologous Proteins

To characterize the evolution of the *D. nobile* DnAGL19 protein, an alignment and phylogenetic analysis was performed using DnAGL19, DOSOC1, and homologous protein sequences from species for which a whole genome sequence was available. Two lines of evidence from these analyses indicated that DnAGL19 is orthologous to members of SOC1/TM3-like subfamily in eudicot and monocot grass. First, the DnAGL19 protein contains the typical organization of the MADS-Box, K domains, and importantly, the characteristic SOC1-motif at the C-terminus, confirming that DnAGL19 is a SOC1/TM3-like protein (**Figure 2**, Supplementary Figure S3) (Vandenbussche et al., 2003). Second, DnAGL19 was grouped in a cluster with other members of SOC1/TM3-like subfamily from angiosperm plants, which is distant from the sister cluster of AGL6-like proteins in the NJ tree (Supplementary Figure S2), indicating that DnAGL19 has the common recent ancestor(s) with the SOC1/TM3-like proteins. However, the NJ tree further indicated that DnAGL19 and DOSOC1 clustered together as an orchid-specific branch that is independent from the eudicot and monocot grass branches (**Figure 1**), suggesting functional divergence of orchid SOC1/TM3-like proteins from their orthologs.

Divergence of DnAGL19 from its orchid ortholog, DOSOC1, was supported by the fact that amino acid substitutions comprised the major variation between these two proteins (**Figure 2**, Supplementary Table S3), and that the substitutions were rare in DOSOC1 and other orthologs. This suggests that DnAGL19 has a diverged biological function from DOSOC1, such as regulating flowering, probably including other development processes, via distinct pathways. Indeed, overexpression of *DnAGL19* did not perturb the expression of *LFY* in transgenic *A. thaliana*, which differs from the observation that *DOSOC1* promotes flowering in *DOSOC1* expressing *A. thaliana* by upregulating *LFY* and *AGL24* (Ding et al., 2013). As shown in Supplementary Table S3, expression of *FT* and *HOS1* were differentially regulated in the *DnAGL19* overexpressing lines depending on temperature cues. However, it is not known whether these two genes are influenced by DOSOC1, and future studies will address whether the differences between DnAGL19 and DOSOC1 contribute to variation in flowering and cold-tolerance in different *Dendrobium* species.

### DnAGL19 Regulates Flowering via Pathway Mediated by AP1 Ortholog

The expression of the *A. thaliana AP1* gene was significantly activated at an earlier time point in the *DnAGL19* overexpressing lines than in wild type plants (**Figure 5**, Supplementary Figure S6). This would tend to promote the transition from vegetative to reproductive development and/or the establishment of floral organ identity (Mandel et al., 1992). However, it should be noted that the acceleration of flowering in the overexpression lines was weak (<2 days earlier flowering, no decrease in RL number; **Table 1**). It is known that in *A. thaliana*, SOC1 upregulates *AP1* expression via a pathway mediated by *LFY* or *FT* (Liu et al., 2008; Kaufmann et al., 2010; Lee and Lee, 2010; Benlloch et al., 2011). However, the expression of *LFY* was not altered by overexpression of *DnAGL19* (**Figure 5**, Supplementary Figure S6), indicating that *LFY* is not a component of the *DnAGL19-AP1* module. The expression of *FT* was down-regulated in the *DnAGL19* overexpressing lines (**Figure 5**), indicating that suppression of *FT* by DnAGL19 did not cause the *AP1* activation. On the contrary, it is possible that the suppression of *FT* expression in combination with *DnAGL19* overexpression may have had the reverse effect on flowering and weakened the consequences of *AP1* activation. Thus, we conclude that overexpression of *DnAGL19* in *A. thaliana* had opposite effects on *AP1* and *FT* expression which counteracts each other and results in weak promotion of flowering.

For the purpose of discovering the regulation of the downstream targets of *DnAGL19* in *D. nobile* in the future, the *D. nobile AP1* ortholog was identified (data not shown). Overexpression of the *D. nobile AP1* ortholog in *A. thaliana* not only promoted floral transition but also altered leaf and petal shape (Supplementary Figure S5), indicating that this AP1-like protein has conserved functions to its *A. thaliana* ortholog (Mandel et al., 1992).This information will be helpful in identifying a genetic link between *DnAGL19* and the *D. nobile AP1* ortholog.

### DnAGL19 Regulates the Activity of the *HOS1-FT* Module

Flowering is mediated by complex GRNs, with floral regulators modulating each other’s expression through various feedback-loops (Pose et al., 2012). *A. thaliana* SOC1 plays a central role during flowering regulation, controlling the activity of a large number of downstream genes (Lee and Lee, 2010; Immink et al., 2012). FT was previously proposed to form a feedback loop with SOC1 in *A. thaliana*, where SOC1 would compete with SHORT VEGETATIVE PHASE (SVP) protein for common targets, such as *AP2*-like or *TEM*-like genes, to alter the expression of *FT* and therefore flowering (Tao et al., 2012), or to up-regulate the expression of *FT* in leaf phloem (Wang et al., 2009). In the present study, we observed that under normal LD conditions, *FT* expression was substantially suppressed by the SOC1/TM3-like protein, DnAGL19 (**Figure 5**). It is not clear whether this DnAGL19-associated FT suppression is or not in relation to SVP or its downstream targets, but it likely occurs through activation of *HOS1* (**Figure 5**) which has been reported to repress *FT* and activate *FLC* under cold stress (Lazaro et al., 2012; Jung et al., 2013; Jung and Park, 2014). Loss of *HOS1* function has been shown to lead to an up-regulation in *FT* expression (Lazaro et al., 2012), which is consistent with our data, meaning that the activation of *HOS1* by overexpression of *DnAGL19* ultimately results in a delay in floral transition. The HOS1 protein has previously been demonstrated to mediate the degradation of the CO protein, thereby repressing *FT* transcription (Jung et al., 2012; Lazaro et al., 2012). It is reasonable to conclude that *DnAGL19* is involved in the *HOS1-CO-FT* module to regulate the flowering time in the transgenic *A. thaliana* lines. Although *AP1* expression was activated earlier in the *DnAGL19* overexpressing lines (**Figure 5**), this was not sufficient to overcome the negative regulation of flowering by the up-regulation of *HOS1*.

**FIGURE 6:**
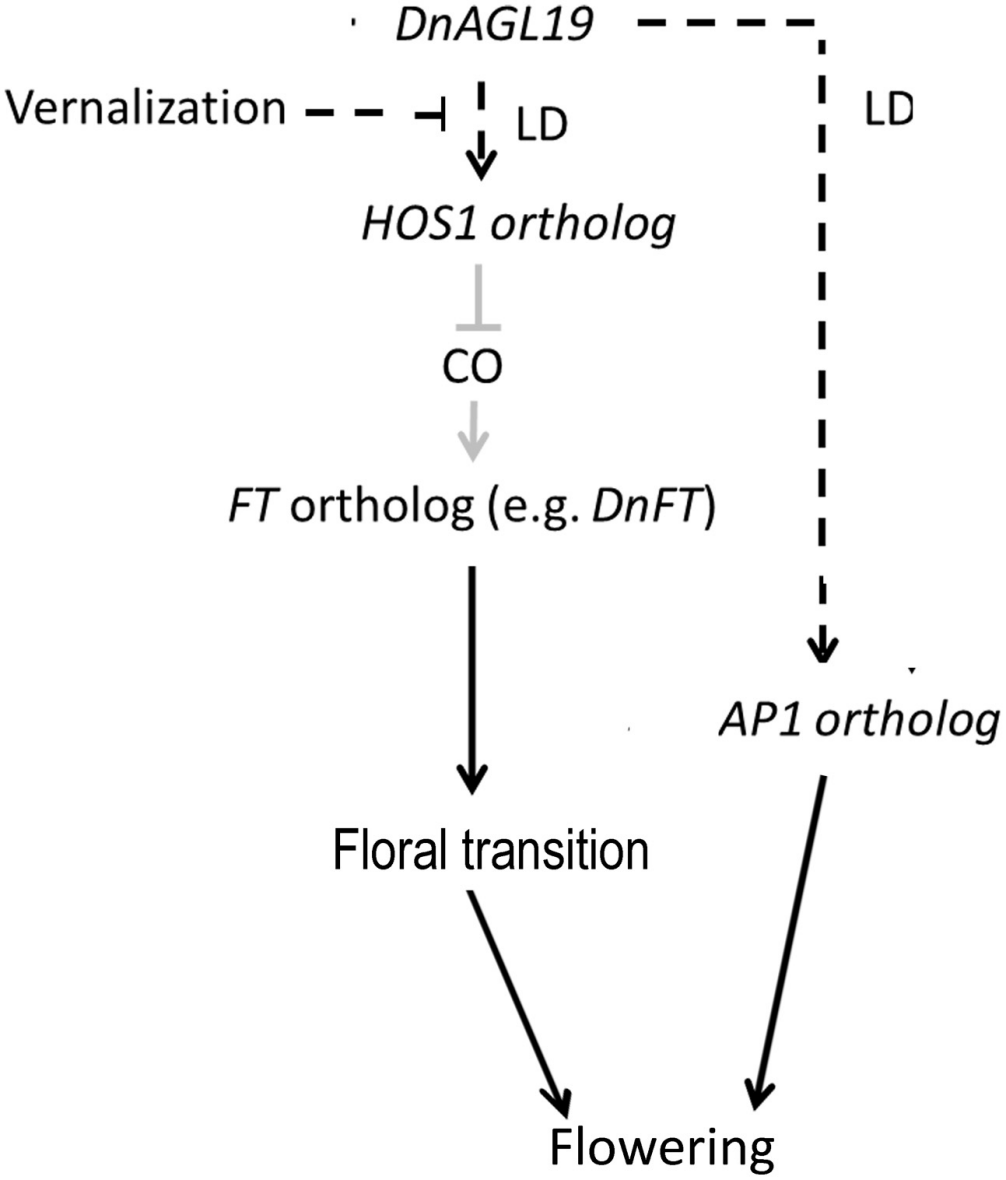
**A predicted model of the gene regulation network (GRN) involving *DnAGL19* to control *D. nobile* flowering**. High levels of DnAGL19 expression regulate flowering via *FT*-dependent and *AP1*-dependent pathways which are independent from each other. In the *FT*-dependent pathway, the *HOS1-CO* module responds to the environmental temperature cues to differentially regulate the expression of *FT* ortholog. Under conditions with higher environmental temperature (e.g., in spring), expression of *HOS1* is activated by DnAGL19 and therefore results to down-regulation of *FT* ortholog probably duo to degradation of CO protein. In winter with long-term low temperature, the activation of *HOS1* is suppressed and the *FT* ortholog is up-regulated, by which floral transition is promoted. On the other hand, activated expression of DnAGL19 also results in up-regulation of *AP1* ortholog to promote the establishment of floral meristem identity, but the components upstream and/or downstream the *AP1* ortholog remains to be clarified. The black solid lines indicate that the *D. nobile* orthologs of *FT* and *AP1* have been verified to be functional in flowering regulation. The *HOS1-CO-FT* module (shown with gray solid lines) has been identified in *A. thaliana* (Jung et al., 2012; Lazaro et al., 2012) but has yet to be characterized in *D. nobile*. The black dashed lines show the regulations observed at the transcriptional level.

*FLOWERING LOCUS C* gene is also a regulation target of HOS1 protein. HOS1 acts as a competitor of HDA6, binding to *FLC* chromatin and removing HDA6 to activate *FLC* in a FVE-dependent manner (Jung et al., 2013; Jung and Park, 2014). But the FLC expression was barely altered in *DnAGL19* overexpressing lines. On the other hand, a *FLC* ortholog has not been identified from *D. nobile* or other phylogenetically closely related orchid species until now (Liang et al., 2012; Cai et al., 2015). We thus propose that the DnAGL19-HOS1-FT module may be the main GRN in *D. nobile.* Transcriptional suppression of *DnFT* was previously observed to occur in parallel with an increase in DnAGL19 transcript levels in *D. nobile* axillary buds during vernalization (Li et al., 2012; Liang et al., 2012), and this is in agreement with the co-expression of *DnAGL19* and *A. thaliana FT* in the present study. This suggests a link between *DnAGL19* and the *FT* ortholog, at least in the axillary buds of adult *D. nobile*, and the HOS1 homolog may act as an intermediary component, as discussed above. Furthermore, an *AP1* ortholog has also been shown to be co-activated with *DnAGL19* in axillary buds (Liang et al., 2012). Thus, DnAGL19-mediated repression of *DnFT* and activation of *DnAP1* may contribute to the regulation of *D. nobile* floral bud development (**Figure 6**).

### Vernalization Regulates Flowering in Combination with DnAGL19

It is well-known that vernalization promote flowering in *A. thaliana*, wheat, and other species. SOC1/TM3-like proteins, such as *A. thaliana* SOC1 and AGL19, function in vernalization-related pathways (Alexandre and Hennig, 2007; Lee and Lee, 2010). It has also been reported that intermittent cold temperatures delay flowering in *A. thaliana*, which is associated with HOS1-mediated repression of *FT* and activation of *FLC* (Jung et al., 2012; Lazaro et al., 2012; Jung and Park, 2014). Vernalization has no effect on *HOS1* expression in wild type plants (Lee, 2012). In our study, however, the prolonged low-temperature treatment (i.e., vernalization for 4 weeks at 4°C) imposed on the *DnAGL19* overexpressing plants resulted in a down-regulation of *HOS1* expression (**Figure 5**). This suggests that high level of DnAGL19 has negative effects on *HOS1* expression by combination with vernalization. Under the same conditions, *FT* transcription was activated (**Figure 5**), which may be attributed to the down-regulation of *HOS1*. Although elevated expression of *DnFT* as a consequence of vernalization has previously been observed in *D. nobile* leaves (Li et al., 2012), its possible co-expression with DnAGL19, or other SOC1/TM3-like orthologs, following vernalization has not been established. We propose that high levels of *DnAGL19* expression in leaves (**Figure 3**) may explain the *DnFT* activation in leaves under these conditions. We also suggest that DnAGL19 may couple the long-term low-temperature signal to the inhibition of *HOS1* expression, resulting in an up-regulation of *FT* expression via the HOS1-CO-FT module (**Figure 6**). However, additional studies of the genetic linkage between *DnAGL19* and *DnFT* are needed to verify this hypothesis.

Our results suggest the existence of a GRN in which the SOC1/TM3-like ortholog, DnAGL19, regulates *FT* expression through the intermediary protein HOS1 (**Figure 6**). The *DnAGL19-HOS1-FT* module is likely differentially regulated, depending on whether or not the plant is experiencing vernalization conditions. Under long-term low temperature such as during winter, high level of *DnAGL19* might repress the expression of the *HOS1* ortholog, allowing the activation of *FT* and the initiation of floral transition. Subsequently, in the spring, DnAGL19 would then up-regulate *HOS1* expression to repress *FT* in the new warmer conditions and *AP1* is activated at the same time, promoting flower development. Although additional evidence is needed to confirm this hypothesis, this GRN would explain the observed features of flowering and adaption in *D. nobile*. The results presented here, shed light on the mechanisms underlying flowering control in Nobile-type *Dendrobium*.

## Author Contributions

X-RL, TP and W-QL generated the *DnAGL19*-overexpressed *Arabidopsis* and worked on phenotypic analysis and transcription detection, LG contributed to the generation and phenotypic analysis of *DnAP1*-overexpressing *Arabidopsis*. X-JW and H-QL contributed to experimental design and manuscript revision. SL designed the experiments, performed data analysis and wrote the manuscript.

## Conflict of Interest Statement

The authors declare that the research was conducted in the absence of any commercial or financial relationships that could be construed as a potential conflict of interest. The reviewer (Dr. Jun-Jun Liu) and Handling Editor declared their shared affiliation, and the Handling Editor states that the process nevertheless met the standards of a fair and objective review.

## Footnotes

1 http://www.siamnobile.com/aboutnobile.html

2 http://phytozome.jgi.doe.gov/pz/portal.html

3 http://www.ncbi.nlm.nih.gov/

4 http://www.ch.embnet.org/software/BOX\_form.html

5 http://weblogo.berkeley.edu/logo.cgi
