# Supplementary material for: Overexpression of an Orchid (Dendrobium nobile) SOC1/TM3-Like Ortholog, DnAGL19, in Arabidopsis Regulates HOS1-FT Expression
Source: Front Plant Sci. 2016 Feb 9;7:99. doi: 10.3389/fpls.2016.00099 (PMC4746357; doi:10.3389/fpls.2016.00099)

Table S1. Primers used in this study.

Taget gene	Primer pair (5' → 3')	Note
D. nobile		
DnAGL19	cgctctagagATGGTGAGGGGAAGGACGG	for 35S::DnAGL19-6myc construction
	cgcctcgagCAGGCCATCTACATACAGTTCGG	
DnAGL19	CTCTGCTGTGCCTAAATGCT	RT-PCR; real-time qPCR
	AACTGTTGGGTTCTTCCTCC	
18S rRNA	GGTTCGCTGCTCGTGACTCT	RT-PCR; real-time qPCR
	CAGGCACCGCTTATTTTTACA	
Arabidopsis		
ACTIN 2/7	CAGTGTCTGGATCGGAGGAT	real-time qPCR
	TGAACAATCGATGGACCTGA	
FT	AATTGTCAGAGGGAGAGTGGC	real-time qPCR
	TAGGCATCATCACCGTTCGT	
FLC	TAGCCACCTTAAATCGGCGG	real-time qPCR
	TACAAACGCTCGCCCTTATCA	
SOC1	GCTGAAGTTTCTCTTATCATC	real-time qPCR
	CCCAATGAACAATTGCGTCTC	
HOS1	GCACAAGGATGCAACCAGAC	real-time qPCR
	ATCTGACCGCCATCTCAATC	
LFY	GCTAAAGACCGTGGCGAA	real-time qPCR
	GCATCCACCACGTCCAGA	
AP1	ATGGGAAGGGGTAGGGTTCAATTG	real-time qPCR
	ATGCTGTTTTGCTCCTGTATGG	

Table S2. Segregation ratio of Kanamycin resistance at T1 generation of 8 putative 35S::DnAGL19-6myc transgenic lines. Kanamycin (50 μg/mL) was added to the MS plate for resistant plant screening.

Line No.	Number of resistant plants	Number of non-resistant plants	Segregation ratio
Col	0	40	0
35S::DnAGL19-6myc			
#1	33	9	3.7 : 1
#2	34	22	1.6 : 1
#3	67	24	2.79 : 1
#4	144	56	2.57 : 1
#5	33	4	8.25 : 1
#6	48	6	8 : 1
#9	23	9	2.5 : 1
#10	108	3	36 : 1

Table S3. Differences in peptide sequences and downstream targets of DnAGL19 from DOSOC1 and AtSOC1/consensus. Differences in amino acid sequences (I) and the regulations of putative targets (II) are shown. The symbol "_" indicates a "Gap" at the corresponding position shown in Figure 2D. The numbers indicate the position on DnAGL19 protein. LD: long-day photoperiod; V: vernaliztion; up/down: expression of target gene being activated/repressed.

I. Amino acid substitutions:

	DnAGL19	DOSOC1	Consensus
**MADS box**	R_5	K	K
	E_{40}	D	D
**K domain**	A_{103}	V	L
	I_{131}	L	L
	Q_{133}	E	R
**SOC-1 motif**	D_{210} (Inserted)	_(Gap)	_(Gap)
	L_{212}	W	L
	$\_(Gap)_{213}$	P	P

II. Downstream targets and the regulation by SOC1 ortholog:

	DnAGL19	DOSOC1	AtSOC1
**AP1**	Up	Up (Ding et al., 2013)	Up (Liu et al., 2008)
**LFY**	Not changed	Up (Ding et al., 2013)	Up (Liu et al., 2008)
**FT**	Down (LD)<br>Up (V +LD)	Not determined	Up/Down in prediction (Tao et al., 2012); not changed (Yoo et al., 2005)
**HOS1**	Up (LD)<br>Down (V+LD)	Not determined	-

Ding, L., Wang, Y., and Yu, H. (2013). Overexpression of DOSOC1, an ortholog of Arabidopsis SOC1, promotes flowering in the orchid Dendrobium Chao Parya Smile. *Plant and cell physiology* 54**,** 595-608.

Liu, C., Chen, H., Er, H.L., Soo, H.M., Kumar, P.P., Han, J.H., Liou, Y.C., and H., Y. (2008). Direct interaction of AGL24 and SOC1 integrates flowering signals in Arabidopsis. *Development* 135**,** 1481-1491.

Tao, Z., Shen, L., Liu, C., Liu, L., Yan, Y., and Yu, H. (2012). Genome-wide identification of SOC1 and SVP targets during the floral transition in Arabidopsis. *Plant J* 70**,** 549-561.

Yoo, S.K., Chung, K.S., Kim, J., Lee, J.H., Hong, S.M., Yoo, S.J., Yoo, S.Y., Lee, J.S., and Ahn, J.H. (2005). Constans activates suppressor of overexpression of constans 1 through Flowering Locus T to promote flowering in Arabidopsis. *Plant Physiology* 139**,** 770-778.

Figure S1. Phylogenetic tree adopted from Phytozome 10.2. Species included into this study are framed.

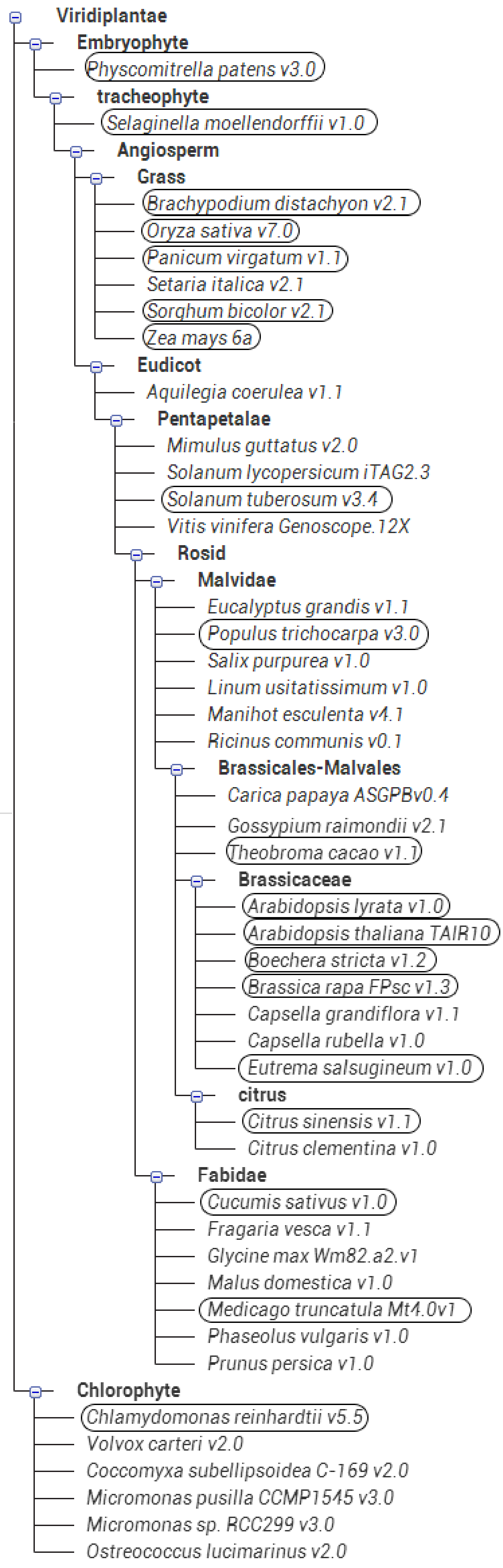

Figure S2. Phylogenetic analysis for DnAGL19 and its homologs. Bootstrap NJ tree was constructed and rooted by a MADS-box containging protein in *C_reinhardtii* . SOC1/TM3-like orthologs from *Arabidopsis* (blue), rice (green) and *Dendrobium* (red) are indicated.

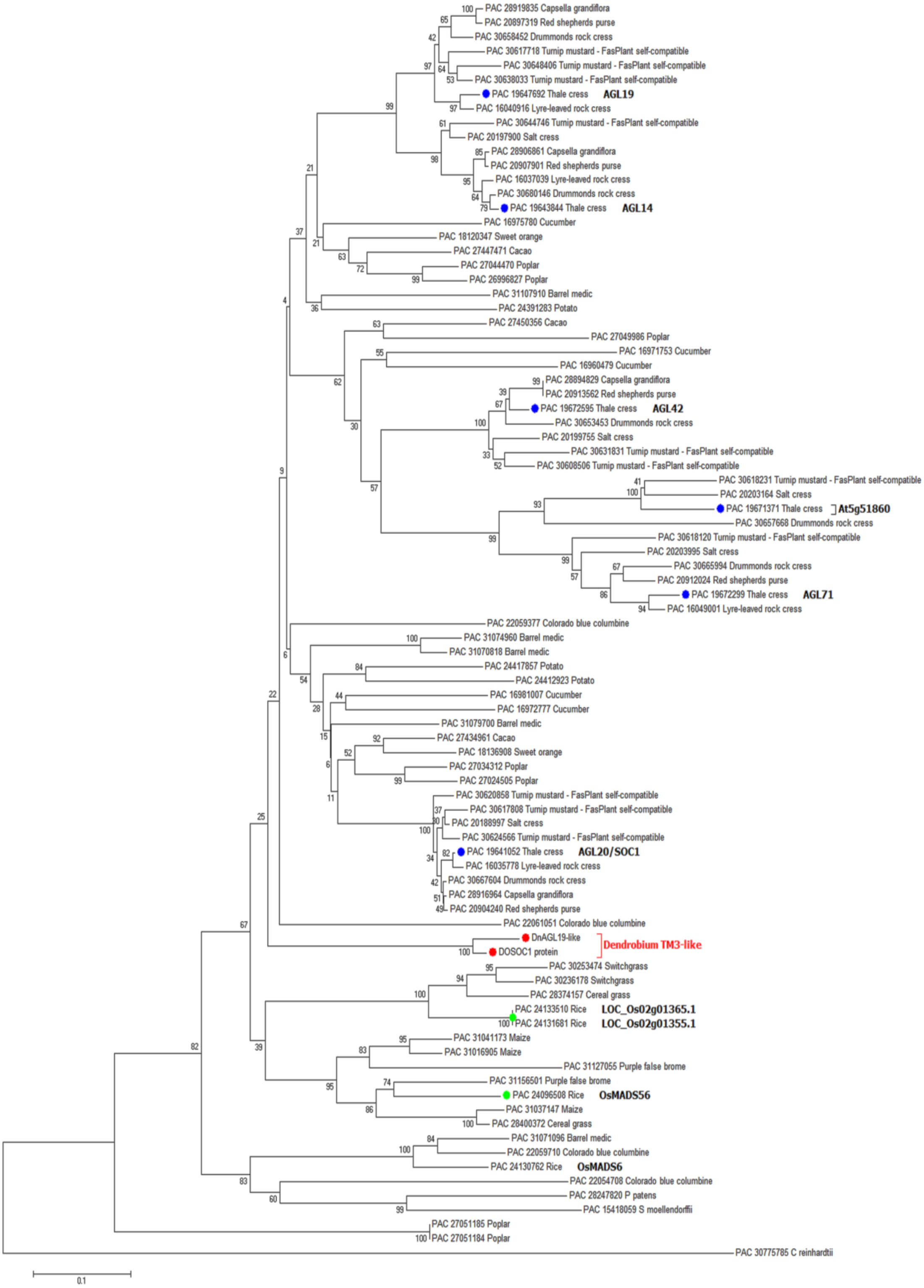

Figure S3. Alignment of DnAGL19 to the orthologs from *A. thiliana*, rice, *Dendrobium* Chao Parya Smile and some other species. The positions with amino acid substitution between DnAGL19 and DOSOC1 are indicated by red upward arrows in MADS and K domains and is framed in red in SOC1-motif.

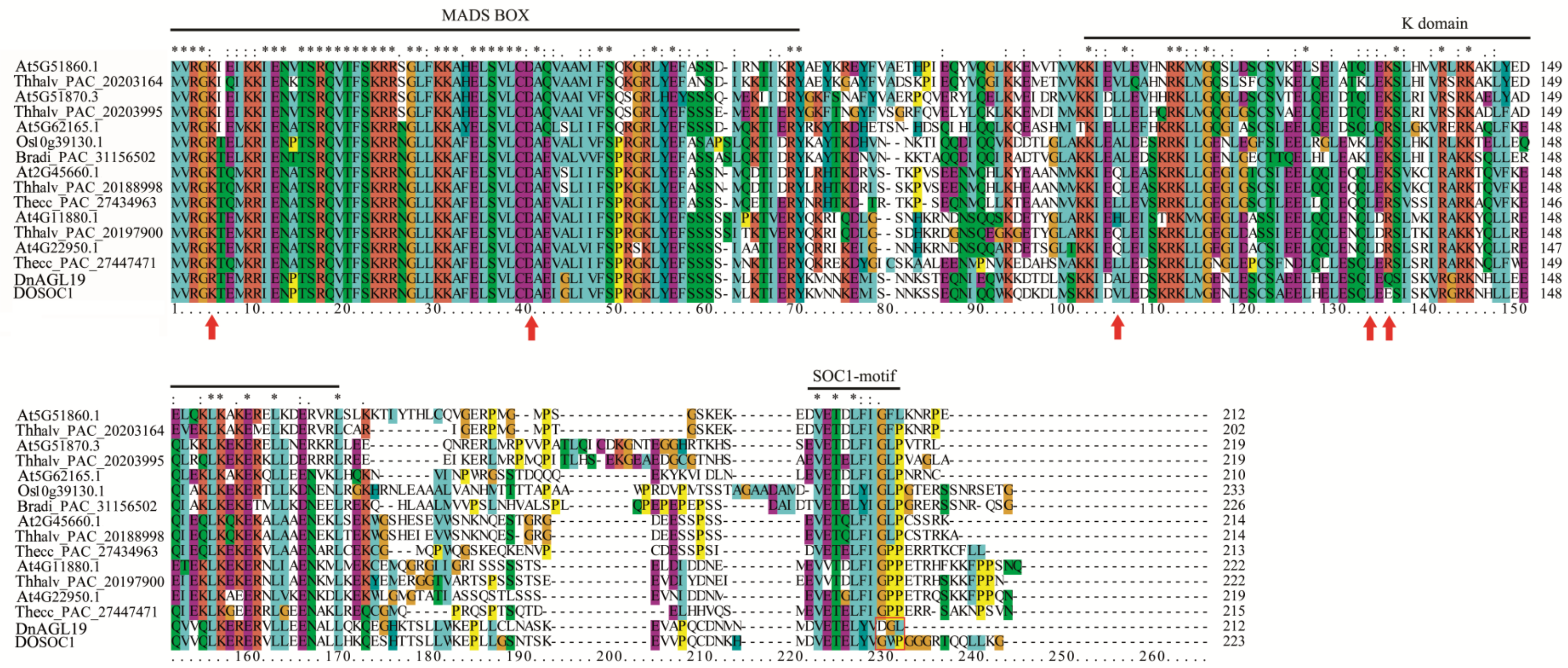

Figure S4. Distribution of DTB of DnAGL19-overexpressing plants at T2 generation. About ten plants generated from each DnAGL19-overexpression lines were analyzed. The DnAGL19 insertion was verified by PCR at T2 generation and those containing the insertions were included to evaluate the flowering phenotypes. Student t-test was applied to each dataset to evaluate the significance of difference. "**" indicates *p*-value < 0.01. N: non-vernalization; V: vernalization. Error bars show the values at 2.5% and 97.5% percentile.

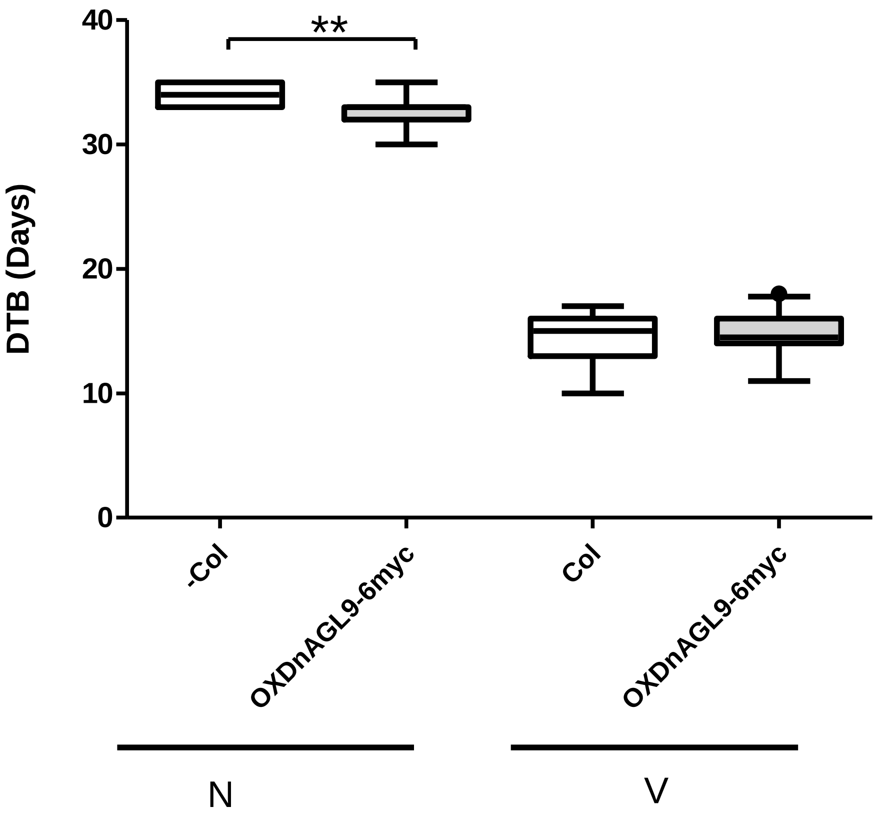

Figure S5. Phenotypes of transgenic Arabidopsis overexpressing *D. nobile AP1-like* ortholog. (A) Distribution of the number of rosette leaves (RL) at T1 generation. (B) Growth of three transgenic lines under normal LD condition. (C) Development of line # 52 at different stages. The enlarged image of inflorescence is shown.

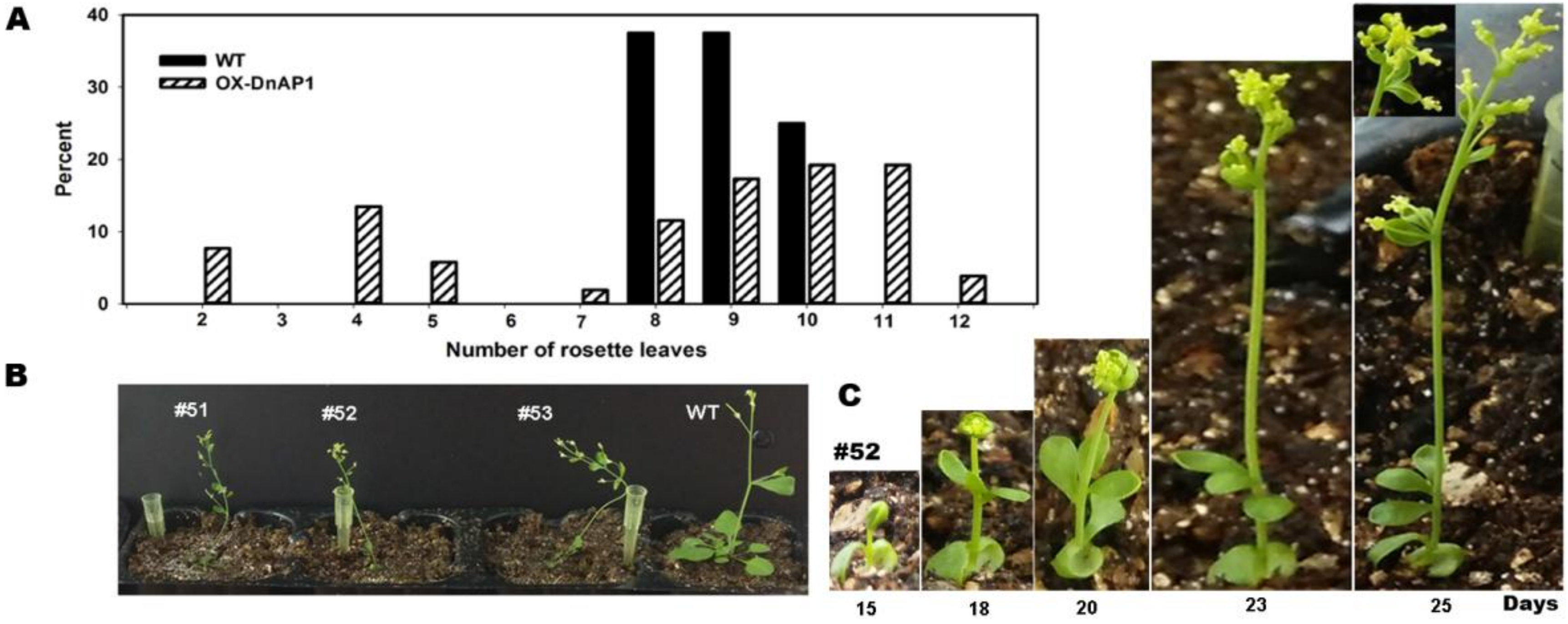

Figure S6. Expression of *AP1* and *LFY* in *DnAGL19*-overexpressing Arabidopsis and wild type. qPCR results are shown. Error bars indicate SD. ** *p*-value < 0.01.

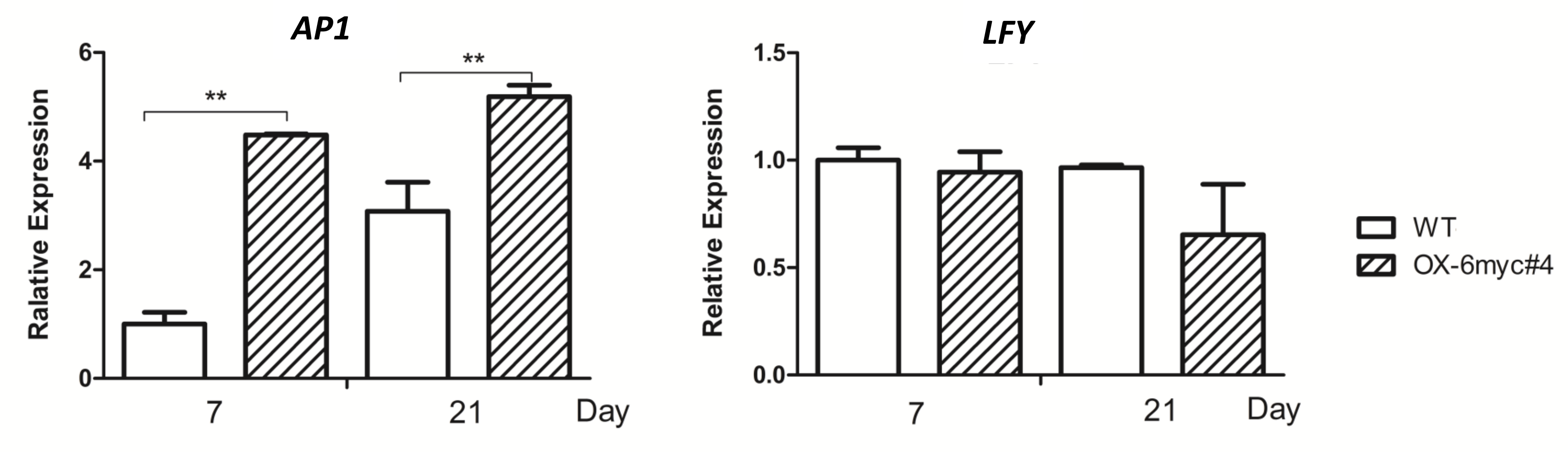

Figuer S7. Expression of *HOS1* and *FT* in DnAGL19-overexpressing line #9. Data are shown in mean + SD. * indicates the *p*-value < 0.05.

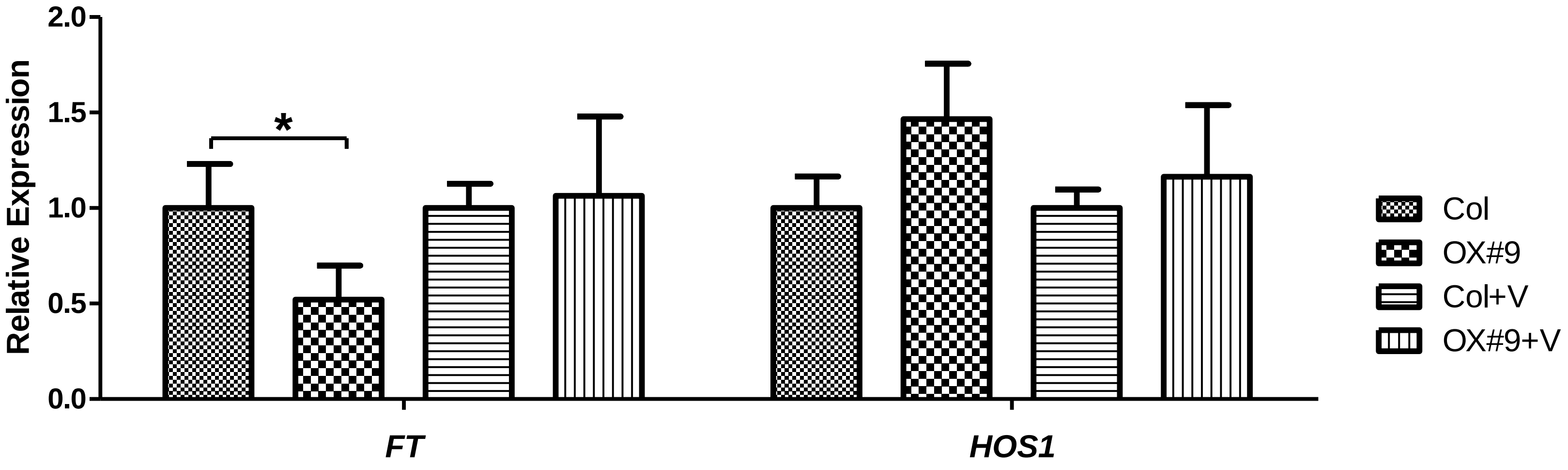

Supplement: Supplementary file 1 [file Presentation_1.PDF]
